# Bortezomib advanced mechanisms of action in multiple myeloma, solid and liquid tumors along with its novel therapeutic applications

**DOI:** 10.17179/excli2022-5653

**Published:** 2023-01-16

**Authors:** Mohammad Alwahsh, Joviana Farhat, Shahd Talhouni, Lama Hamadneh, Roland Hergenröder

**Affiliations:** 1 Department of Pharmacy, Faculty of Pharmacy, Al-Zaytoonah University of Jordan, P.O. Box 130, Amman, 11733, Jordan; 2 Leibniz-Institut für Analytische Wissenschaften-ISAS-e.V., 44139 Dortmund, Germany; 3 Institute of Pathology and Medical Research Center (ZMF), University Medical Center Mannheim, Heidelberg University, 68167 Mannheim, Germany; 4 Department of Epidemiology and Population Health, College of Medicine and Health Sciences, Khalifa University, Abu Dhabi, PO Box 127788, United Arab Emirates

**Keywords:** bortezomib, proteasome inhibitor, multiple myeloma, solid tumors, liquid tumors

## Abstract

Bortezomib (BTZ) is a first-in-class reversible and selective proteasome inhibitor. It inhibits the ubiquitin proteasome pathway that leads to the degradation of many intracellular proteins. Initially, BTZ was FDA approved for the treatment of refractory or relapsed multiple myeloma (MM) in 2003. Later, its usage was approved for patients with previously untreated MM. In 2006, BTZ was approved for the treatment of relapsed or refractory Mantle Cell Lymphoma (MCL) and, in 2014, for previously untreated MCL. BTZ has been extensively studied either alone or in combination with other drugs for the treatment of different liquid tumors especially in MM. However, limited data evaluated the efficacy and safety of using BTZ in patients with solid tumors. In this review, we will discuss the advanced and novel mechanisms of action of BTZ documented in MM, solid tumors and liquid tumors. Moreover, we will shed the light on the newly discovered pharmacological effects of BTZ in other prevalent diseases.

## Introduction

Proteasome is a large protein complex responsible for intracellular proteolysis stabilization and regulation of the protein quality control by destroying misfolded or aggregate-prone damaged protein (Collins and Goldberg, 2017[28]; Davis et al., 2021[32]; Majumder and Baumeister, 2019[95]; Mao, 2021[98]). Proteasome is also used by eukaryotic cells to maintain myriad cellular pathways by a gradual key proteins excretion mechanism (Bard et al., 2018[8]; Becker et al., 2019[12]; Dong et al., 2019[36]; Fritze et al., 2020[42]; Majumder et al., 2019[96]). Specifically, 26S proteasome is responsible for the cytoplasmic, nucleic and superficial proteolysis of approximately all organelles (Ding et al., 2017[34], 2019[35]; Worden et al., 2017[160]). Within the 26S proteasome, a 19S regulatory particle (RP) exhibits the degradation signal and unfolding process of the target protein-substrate (Wehmer et al., 2017[158]; Zhu et al., 2018[176]). The 20S core particle (CP) constitutes the catalytic core of the 26S proteasome which dissociates the unfolded polypeptide into shorter peptides or amino acids (de la Peña et al., 2018[33]; Mendes et al., 2020[102]). However, the free 20S proteasome complex is capable of retaining basal proteolytic activity for substrates with an unstructured or unfolded stretch (Boughton et al., 2020[17]; Clague et al., 2015[27]; Longworth and Dittmar, 2019[92]; Oh et al., 2018[119]; Yau et al., 2017[167]).

In the mid-1990s, Adams et al. thought that if the proteasome was responsible for eliminating damaged or harmful proteins in the cell, it might also be removing beneficial ones which originally prevent the acquisition of complex diseases such as cancer (Wang et al., 2017[157]). Consequently, experimental studies have proved the close connection between proteasome dysfunction and cancer disease progression (Schweitzer et al., 2016[133]). This pathophysiological association helped researchers to develop an entirely new class of anticancer drugs called proteasome inhibitors such a bortezomib (BTZ) (Jakubowiak et al., 2011[64]). 

BTZ was originally known as PS-341 and developed by ProScript, a biotech company, to treat muscle weakness and muscle loss associated with AIDS and muscular dystrophy (Jakubowiak et al., 2009[65]). The National Cancer Institute (NCI) assessed if PS-341 could be effective in a variety of cancer cell lines (Niu et al., 2021[117]). Researchers found that PS-341 significantly reduced cell growth in many different types of cancer cells by slowing down the action of the proteasome leading to the destruction of key proteins that help cells fight off cancer (Hallberg and Palmer, 2013[52]). BTZ was seen to potentially limit the chymotrypsin-like proteasomal activation by reversibly interacting with the β5 subunit of the 20S proteasome leading to a complete inhibition of proteasomal activity followed by the accumulation of poly-ubiquitinated proteins in cells (Yau et al., 2015[166]). In 2003, US FDA approved BTZ for the treatment of relapsed and refractory Multiple Myeloma (MM) (Adams, 2014[1]). MM is an incurable cancer of the blood that affects approximately 14,000 patients in the US, annually (Adams, 2014[1]). BTZ was also applied in relapsed mantle cell lymphoma and diffuse large B-cell lymphoma (Vesole et al., 2015[154]). In 2005, US FDA fully approved BTZ use as a second-line MM therapy, and as a first-line therapy for patients with newly diagnosed MM after only three years (Korde et al., 2015[77]). Currently, BTZ is being studied for use in a wide variety of blood cancers and solid tumors (Richardson et al., 2014[129]).

In the present review, we will discuss the recently discovered advances of BTZ mechanisms of action in MM, solid and liquid tumors and diseases other than cancer. 

## Advanced Mechanism of Action of BTZ in MM

### Intrinsic pathways activation

Calreticulin (CALR) is a chaperone protein found in the endoplasmic reticulum (ER), the cytoplasm, and at the outer surface of the cell. CALR is involved in the proper folding of newly formed proteins and maintenance of calcium ions levels and gene activity control, stabilization of cell growth and division, proliferation, migration and adhesion mechanisms in addition to the regulation of programmed cell death known as apoptosis (Gold et al., 2010[46]; Malcovati et al., 2014[97]; Michalak et al., 2009[106]). CALR pathway activation constitutes the base of a successful BTZ treatment for MM cells. Indeed, CALR processing is initiated through the phosphorylation of eukaryotic initiation factor 2α (eIF2α) by the ER stress kinase eIF2α kinase-3 (EIF2AK3), best known as PRKR-like endoplasmic reticulum kinase (PERK) which induces an integrated response stress (Hopfner and Hornung, 2020[56]; Nangalia et al., 2013[114]). In response to chemotherapy inducers such as BTZ, this phosphorylation event occurs downstream of a general inhibition of DNA-to-RNA transcription. High-dose BTZ is proved to reduce global RNA synthesis in osteosarcoma cells, suggesting that this can also occur in MM cells (Kroemer and Zitvogel, 2021[78]). However, this hypothesis is subject to further investigations.

Moreover, BTZ is characterized by immune-stimulatory effects which express heat shock protein 90 on MM cells, easing their recognition by dendritic cells (DCs) (Spisek et al., 2007[139]). Thus, intracellular proteins will flow toward the cell surface, marking the Immunogenic Cell Death (ICD). Consequently, CALR of the endoplasmic reticulum ER is released from the ER lumen to the plasma membrane surface. There, CALR initiates the phagocytosis of dying cells by DCs through the “eat-me” signal. This CALR-dependent ICD emphasized on BTZ ability to drive CALR toward the surface of human or mouse MM cells leading to the phagocytosis of these cells (Gulla et al., 2021[50]). Hence, achieving an optimal therapeutic efficacy of BTZ against MM cells is suggested to depend on CALR activation and the patient's immune system. *In vivo*, MM cells were only responsive to BTZ treatment in the immunocompetent setting rather than in immunodeficiency state. But CALR deterioration in MM cells limited the efficacy of BTZ against MM tumors even in immunocompetent mice (Galluzzi et al., 2017[43]). Also, when BTZ-killed murine MM cells were injected into immunocompetent mice, MM cells were unable to grow 1 week post-injection (Galluzzi et al., 2017[43]). This vaccination effect was reduced when the CALR gene was inactivated in MM cells. 

In addition to CALR, STING pathway also reflected a major factor behind cancer cells responsiveness toward BTZ. STING is responsible for cell death induction and release of cancer cell antigens. STING activation has been proved to enhance cancer antigen presentation, contribute to the priming and activation of T cells, and facilitate the trafficking and infiltration of T cells into tumors in order to kill cancer cells (Zhu et al., 2019[175]). In fact, STING drop-out prevented the activation of IFNA1, IFNB1, and CXCL9 gene transcription by BTZ and abolished the capacity of BTZ-treated human MM cells to stimulate CD4+ EM and CD8+ EM cells in co-cultured immune cells. When combined with a synthetic STING agonist, ADUS-100, BTZ responses were potentiated in a mouse MM model through a fortified infiltration of the tumors by T lymphocytes and an increased phosphorylation of TBK1 *in vitro*. These discussed additive effects were minimized when STING was limited in mouse MM cells or in immune-deficient mice (Montes de Oca et al., 2021[110]). 

So, CALR and STING pathways together account for a complete BTZ pharmacological activity. STING by translocating to the ER, inter-locates with the CALR pathway. Recent trials showed that STING suppression did not influence BTZ-induced CALR exposure. While *in vivo*, CALR knockout prevented the BTZ-mediated induction of interferon stimulated genes in MM mouse model (Tallarida, 2011[144]). The need to focus on the importance of CALR in cytosolic DNA-cGAS-STING-TBK1-type-1 interferon pathway generated a new hypothesis for investigation through the *in vitro* setting. Challenge also apply on specifying the concentration at which CALR deficiency can inhibit this pathway taking into account the potential of cancer cells to escape immune-surveillance by suppressing the CALR exposure pathway at multiple levels (Tallarida, 2011[144]). MM cells can succeed to develop such pathway deficiencies as a result of immune-selection, particularly after recurrence of cancer following BTZ-based therapeutic regimens.

### Anti-tumor mediators and intrinsic factors expression

Several anti-tumor mediators have been demonstrated to be involved in the control of growth, progression, and dissemination of MM (Kuku et al., 2005[79]). Co-culturing of BTZ-treated human MM cells with DCs and T lymphocytes induced a high release of CD4+ effector memory (EM) cells, CD8+ EM cells and CD8+ T EM cells re-expressing CD45RA (TEMRA). In fact, the production of these immunity boosters was not seen when BTZ treated MM cells were removed from the co-culture or when BTZ alone was added to the co-culture of DCs and T cells (Zitvogel et al., 2016[178]). 

Concomitantly, BTZ efficacy was correlated with the activation of particular expressed genes. In CALR-expressing (but not in CALR-deficient) MM, BTZ stimulated 90 immune-related genes that composed the “ICD signature”. Among them, 57 interferon genes responsible for activating a type-1 interferon response mediated by interferon alpha1 (IFNA1), interferon beta1 (IFNB1) and Chemokine (C-X-C motif) ligand 9 (CXCL9, a ligand acting on the receptor CXCR3 on T cells to attract them into the tumor bed) upregulation in *in vitro* MM cells, correlating with the clinical outcome of MM patients treated with BTZ-based regimens (Galluzzi et al., 2020[44]). This type-1 interferon response following BTZ administration caused a high release in cytosolic DNA in the form of micronuclei, accumulation of the cytosolic DNA sensor cyclic GMP-AMP synthase (cGAS), activation of transmembrane protein 173 (TMEM173, best known as STING) and activation of TANK-binding kinase 1 (TBK1) phosphorylation. Data confirms that BTZ is able to induce a type-1 interferon response usually linked to ICD and has been known as “viral mimicry” (Sistigu et al., 2014[136]). Like other ICD-inducing drugs, BTZ stresses and kills MM cells mimicking the viral infection process to the degree that high levels of type-1 interferons are activated. Recently, BTZ exhibited its potential to upregulate a key cellular miRNA involved in T cell function known as miR-155. This activation resulted in downregulating of BTZ targets which are cytokine signaling 1 (SOCS1) and inositol polyphosphate-5-phosphatase (SHIP1), normally classified as negative endogenous proteins, leading to a suppressed PD-1-mediated T cell exhaustion (Renrick et al., 2021[128]). BTZ confirmed its novel lymphocyte-stimulatory interactions as a crucial factor towards lessening the immunosuppressive actions of tumor on antitumor T cell functions and combining BTZ with other immunotherapies to shrink tumor microenvironment's potential maximally and obtain a full BTZ efficiency (Franchi et al., 2021[41]).

## BTZ Anti-Cancer Efficacy in MM

Novel BTZ mechanisms of action led researchers to evaluate its clinical efficacy when used solely or as a part of an anti-tumor regimen. 

Recent clinical findings proved that MM patients are living longer than those diagnosed 10 years ago because of the availability of new targeted drugs like BZT (Velcade®). In practice, BTZ single therapy prolonged the median overall survival (OS) and the restricted mean survival time (RMST) to a total of 33.9 and 42.9 months, respectively, compared to a lower finding in the chemotherapy group as 27.9 and 38.4 months, respectively (Tutt et al., 2021[150]). However, Velcade® has shown a greater effectiveness when used in combination with two, three, or four other anticancer drugs in patients newly diagnosed with MM. Nowadays, BTZ containing regimens are the guideline treatment option for MM patients. The combination therapy with BTZ, lenalidomide and dexamethasone (VRd) constitutes the first-line regimen for relapsed/refractory as well as newly diagnosed MM in terms of its high impact on overall survival (Ibata et al., 2016[62]). In a three-drug study, 85 percent of patients responded well to the treatment plan whereas 40 percent of patients experienced remission after one to two years. Also, triple use of BTZ, cyclophosphamide and dexamethasone (VCD) achieved an 88 %, 63 % and 89 % of overall response rate (ORR), progression-free survival (PFS) and overall survival, respectively (Choueiri et al., 2021[25]). BTZ, lenalidomide, and dexamethasone VRd marked a greater overall response rate (ORR), progression-free survival (PFS) and overall survival rates of 98 %, 85 % and 95 %, respectively (Choueiri et al., 2021[25]; Okazuka et al., 2020[120]). In BOSTON and OPTIMISM studies, combining BTZ and dexamethasone with **s**elinexor or pomalidomide provided MM patients with 13.93 months and 11.20 months survival free periods following treatment (Parsons et al., 2020[121]; U.S. FDA, 2022[153]). Similar success occurred with a four-drug combination where 96 percent of patients responded to the treatment and 39 percent went into remission (Wakelee et al., 2021[156]). 

In contrast, Cavo et al. proved that hematopoietic stem-cell transplantation (HSCT) in transplant eligible patients can extend patients' PFS to 56.7 months compared to a PFS of 41.9 if those patients will receive BTZ-melphalan-prednisone (VMP) treatment (Cavo et al., 2020[21]). Hence, BTZ used as part of the VRd regimen imposed its greater value as a consolidation therapy, where a 58.9 months' PFS was achieved compared to a 45.5 months' PFS in absence of a consolidated use of BTZ regimen (Cavo et al., 2020[21]). Despite the therapeutic effectiveness of BTZ regimens, patient adherence to a weekly BTZ dose of 1.0 mg/m^2^ for a 48 weeks' treatment period allowed a maximal successful treatment outcome (Ibarra et al., 2021[61]). Sometimes, severe adverse events may occur without regimen's dose reduction during consolidation/maintenance phase. Recent trials approved that at least six 28-day cycles of subcutaneous BTZ (1.3 mg/m^2^ on days 1 and 15), lenalidomide (10 mg on days 1-21) and dexamethasone (40 mg on days 1, 8, 15 and 22) regimen will be associated with an overall response rate and complete response (CR) rate equaling 100 and 43.8 %, respectively. Also, PFS and OS rates at 2.5 years were 66.6 % and 77.3 %, respectively where grade 3 or 4 hematologic or non-hematologic adverse events were not documented (Ibata et al., 2016[62]).

In delayed transplantation scenarios, BTZ also proved its efficacy. VRd regimen prolonged PFS and OS for a total duration of 43 months, 75 months respectively compared to 30 months PFS and 64 months OS values when lenalidomide and dexamethasone (Rd) were used in absence of BTZ. Moreover, a larger fraction of patients in VRd group 16 % (34/216) compared to Rd group 8 % (18/214) achieved a better ORR equal to 82% rather than 72 %, respectively (Durie et al., 2017[38]). Yet, toxicological risks were marked by higher proportions of adverse events of grade 3 or higher reported in (82 %) of patients treated with VRd and (75 %) in patients subject to Rd (Durie et al., 2017[38]). Consequently, researchers formulated a more tolerated version of the RVd known as RVd lite mainly indicated to be used for patients ineligible to transplantation. ORR was achieved by 86 % accompanied with peripheral neuropathy and was reported in 31 (62 %) patients with only 1 patient experiencing grade 3 symptoms (O'Donnell et al., 2018[118]). 

In patients with relapsed and refractory cases of MM, prolonged or enhanced efficacy of BTZ are needed. Remarkably, in multiple myeloma, patients receiving BTZ were able to achieve complete responses associated with longer life expectancy. At the same time, chromosomal mutations were seen to diminish the efficacy of many anticancer drugs and hence worsen cancer patient prognosis and outcome of multiple myeloma treatment such as del (17q13) while del (13q14), amp (1q21), t (4,14), t (14,16) allowed BTZ to overcome poor prognosis (Grosicki et al., 2020[49]). Accordingly, immunoglobulin subtypes IgA and mainly IgG are considered direct biomarkers for driving resistance toward suboptimal response and VRd treatment failure (Keruakous et al., 2021[73]). Bahlis et al. found that venetoclax plus daratumumab/dexamethasone (VenDd) and VenDd with BTZ (VenDVd) produced high rates of durable responses in those patients (Bahlis et al., 2018[7]). BTZ was also evaluated in combination with mitoxantrone, vincristine, pegaspargase and dexamethasone where of the 10 patients enrolled, eight (80 %) achieved a complete remission (CR) or complete remission with incomplete recovery (CRi). In addition to the occurrence of grade 3 or higher infections in four out of 10 patients, and other toxicities commonly associated with BTZ were not seen (August et al., 2020[6]). As BTZ doses are usually administered in cycles, the risk of cellular resistance acquisition in BTZ treated patients remains a major challenge for its clinical usage in practice. Hence, assessing the resistance mechanism of methylome in neuroblastoma cells following BTZ therapy was performed through genome wide methylation process. This cellular shift bypassed the primary anticancer activity of BTZ, thus expressing a proliferative phenotype proportional to the number of treatments administered. Similar cellular effects were not found with lenalidomide treatment nor with non-treated cells cultured under the same experimental conditions. This phenomenon seems to be directly correlated with BTZ treatment (Łuczkowska et al., 2021[93]). Overall, the implementation of new and adjuvant therapies is boosting the trend towards individualizing treatment as well as ameliorating patients' outcomes in case of a high residual risk of recurrence following primary treatment (U.S. FDA, 2018[151], 2020[152]; Sabbah et al., 2020[132]; Tutt et al., 2021[150]; Wakelee et al., 2021[156]). 

## BTZ Implementation in Solid Tumors

BTZ was preliminarily approved for the treatment of MM. Nowadays, BTZ constitutes one of the widely used anticancer agents in other solid tumor types (see Table 1(Tab. 1); References in Table 1: Adams, 2001[2]; Alsahafi et al., 2019[3]; Bashraheel et al., 2020[9]; Benvenuto et al., 2021[13]; Bielskienė et al., 2015[14]; Bray et al., 2018[18]; Carbone et al., 2020[20]; Cerruti et al., 2017[22]; Chow, 2020[26]; Dai et al., 2020[30]; Harsha et al., 2020[55]; Hou et al., 2019[57]; Joshi and Broughman, 2021[68]; Kitamura et al., 2020[75]; Laszlo et al., 2019[80]; Lee et al., 2018[83], 2021[82]; Li et al., 2018[84]; Lo Nigro et al., 2017[91]; Montagnani et al., 2017[109]; Muenchow et al., 2020[111]; Nadhan et al., 2020[112]; Pettersson et al., 2013[122]; Raza et al., 2017[127]; Su et al., 2021[140]; Takács et al., 2020[143]; Taniguchi and Karin, 2018[145]; Tsumagari et al., 2018[149]; Wu et al., 2018[161]; Yang et al., 2018[164]; Zhang et al., 2018[171], 2019[170]).

### Ovarian cancer

BTZ proved its potential to slow the growth and shrink the size of ovarian tumor when combined with an IKK inhibitor named as Bay 117085. BTZ anticancer property was marked through decreased tumor levels of S536P-p65 NFκB in addition to a minimized recruitment to IL-8 promoter in tumor tissues, alleviating levels of IL-8. Further investigations specified that IKK inhibition limits the IL-8 production and potentiates BZ effectiveness in reducing ovarian tumor growth *in vivo* and in turn improving patient response to treatment (Huang et al., 2014[58]). Also, BTZ was seen to grant docetaxel additional effects on ovarian cancer cells through the stabilization of apoptotic proteins like Apaf-1, inhibiting the degradation of cytosolic cytochrome c released by docetaxel, stimulating intrinsic apoptosis and initiating cancer cell death. These favorable mechanisms correlate with a better cellular sensitivity toward docetaxel, leading to a proper chemo-resistance control (Weyburne et al., 2017[159]).

### Bladder cancer

BTZ has been studied in bladder cancer and failed a Stage II clinical trial. Assessment of its response rate, safety, and toxicity, progression-free and overall survival exhibited its ability to be safe yet not a drug of choice as a second line therapy due to the absence of anti-tumor activity (Mirabella et al., 2011[107]; Radhakrishnan et al., 2010[123]). Currently, 3D drug screening in bladder cancer cell lines highlighted BTZ among the “very active” compounds across the 17 bladder cancer cell lines tested, on the basis of its drug sensitivity score 3 (DSS3) (Rajkumar, 2016[124]). So, futuristic application of experimental technologies is needed in order to rate the importance of BTZ possible contribution as a direct therapy in bladder cancer treatment.

### Cervical cancer

Till now, there is no specific study of BTZ on cervical cancer cells but some secondary trials were implemented. In cervical cancer, cells stabilize low levels of intrinsic p53 in function to its rapid proteasomal degradation by E6 and E6-AP proteins (Wustrow et al., 2013[162]). Hence, p53 cellular reactivation mechanism is exhibited either by inhibiting E6 protein at transcriptional and translational levels or through proteasome activity repression by proteasome inhibitors in order to reach an indirect p53 back-up level and potential activity (Soucy et al., 2009[138]; Zhu et al., 2013[177]). Recently, a polyphenolic alkanone, 6-Gingerol (6G), extracted from ginger (*Zingiber officinale Roscoe*) expressed anti-tumorigenic and pro-apoptotic activity against a variety of cancers. Thus, testing its efficacy in cervical cancer was promising. Findings showed its matching mechanism of action with BTZ to stimulate p53 expression along with its target p21. In addition to an increasing release of ubiquitinated proteins in 6G treated cells similar to that of the BTZ treatment. Interacting with proteosomal β-5 subunit was also seen but with a higher affinity than BTZ (Anderson et al., 2015[5]). In parallel, BTZ was able to perform its proteasomal activities in cervical cancer cells mediated by its tumor suppressors such as p53, hDlg and hScrib, especially when combined with cisplatin in another trial. Hence, valorizing the auspicious application of this dual therapy in resistant cervical cancer cases (Singha et al., 2015[135]). Based on this, BTZ efficacy in cervical cancer was marked yet a novel discovered medicine with better physiological characteristics limited its usage.

### Colorectal cancer

Recently, it was proved that targeting poor proteasomal function with radioiodine will limit CT26 colon cancer stem cells resistance to BTZ therapy through multiple mechanisms in terms of sodium-iodide symporter (NIS) fusion protein that usually accumulates in cells with low proteasome activity. From one hand, CT26/NIS-cODC cells exhibited the assembly of NIS and took up of radioiodine under proteasome inhibitory conditions (Mehdizadeh et al., 2021[101]). From the other hand, CT26/NIS-cODC cells enriched for stemness cumulated NIS and executed a high radioiodine uptake. Also, CT26/NIS-cODC cell populations were able to survive following BTZ treatment because of their enrichment with CSCs. Furthermore, an administered 131I treatment strengthened the therapeutic efficacy of BTZ and suppressed cancer stemness along with a high radioiodine uptake *in vivo*. So, 131I therapy was seen to boost the efficacy of BTZ treatment against CT26/NIS-cODC tumors. Concluding that cancer stemness *in vivo* is increased by BTZ alone but is suppressed by adding 131I therapy (Rosenberg et al., 2008[130]). Dual BTZ and epirubicin treatment intensified tumor immunogenicity and the induction of antitumor immunity also. This therapeutic fusion potentiated immunogenic cell death induction of colorectal cancer cells by increasing expression of death receptors such as Fas, which enhanced colorectal cancer cells susceptibility to Fas/FasL mediated tumor cell killing but not healthy non-malignant epithelial cells, to apoptosis. Also, this regimen stabilized the transcriptional activation of Fas in colorectal cancer cells but not in normal cells (Gomez-Abuin et al., 2007[47]). 

### Endometrial cancer

Proteasome inhibitor (PIs) are well known to act on ubiquitin pathways. Recently, an underlying enzyme named as Ubiquitin C-terminal hydrolase L5 (UCHL5) was suggested to be a contributing factor for tumor growth progression and metastasis occurrence in multiple cancer tumors as endometrial cancer (EC). To identify the role of UCHL5 on EC, bioinformatics analysis revealed that UCHL5 overexpression in EC tissues lead to lower overall survival (Merrill et al., 2020[105]). Hence, UCHL5 was seen to be reversibly associated with the 26S proteasome in order to prevent target proteins degradation by hydrolyzing ubiquitin chains (Rastogi and Mishra, 2012[126]). This calls for further investigation of PI therapies that directly target enzymatic signaling pathways including UCHL5. Consequently Pis, mainly BTZ, were administered along with a panel of 23 chemotherapeutic agents and targeted drugs to organoid cultures of seven patients with different histological subtypes of endometrial cancer, including early-stage/grade endometrioid adenocarcinoma and high-grade serous carcinoma. The highest responsiveness was correlated to the combination of a histone deacetylase (HDAC) inhibitor and a proteasome inhibitor. Functionally, HDAC inhibitors and proteasome inhibitors were seen to express a dual proteasome and aggresome blockage resulting in apoptosis due to the accumulation of misfolded proteins (Bruning et al., 2011[19]). In another recent publication, BTZ combined to Histone Deacetylase Inhibitors were able to alleviate the impact of gain-of-function of p53 mutations through an enhanced p53 mutated cancer cell sensitivity towards BTZ caused by the preliminary addition of HDACi (Rastogi et al., 2015[125]). Obtained findings will serve as a crucial foundation for an improved combination therapy to be tested *in vitro* and *in vivo* reaching the main EC endpoints defined through the induction of apoptosis and the endometrial tumor regression. 

### Kidney cancer

In renal carcinoma, the proteasome inhibitor carfilzomib was studied and showed a significant acute and long-term cytotoxicity yet an *in vivo* anti-tumoral activity correlated with the level of accumulation of ubiquitinated proteins (UPs) (Chakraborty et al., 2012[23]). Also, researches tried to evaluate the prognostic role of the ubiquitin proteasome system (UPS) in renal tumors based on the categorization of involved genes. In fact, among 91 differentially expressed UPSs, 48 prognosis related genes were highlighted. These genes can be either subject to downregulation as ASB15, HECW1, RNF150, USP44, FBXO2 or upregulation as MDM4, KCTD13, TRAF2, PCGF1, TRAF3IP2. So, this destabilization of genetic expression marks UPS variations in renal carcinoma (Miyamoto et al., 2013[108]). Such findings impose the need for further studies on the abnormal ubiquitin proteasome system factors' as a main contributor in patient prognosis.

### Nasopharyngeal cancer (NPC)

Nasopharyngeal carcinoma (NPC) reflects one of the complex tumor types since it is related to various environmental and host-dependent factors directly affecting cancerous progression. Majorly, Epstein Barr Virus (EBV) plays a key role in tissue growth, distant metastasis, and immune disability leading to a poor patient prognosis. Therefore, multiple intrinsic factors as EBV related onco-viral proteins such as Latent Membrane Protein family (LMP1, LMP2), Epstein Barr Nuclear Antigen 1 (EBNA1) and EBV-related glycoprotein B (gB) are proved to destabilize signaling pathways reaching a fast metastatic severe presentation of NPC (Meng et al., 2018[104]). Hence, activation signaling of proteasomes seems to be a crucial part of this overall critical mechanism. *In vitro* and *in vivo* investigations implemented the use of proteasome inhibitors in the therapeutic setting of NPC. Recently, Hui et al. tested the efficacy of BTZ combined to SAHA (Vorinostat) in female BALB/c nude (nu/nu) mice. Majorly, various apoptotic mechanisms and effects on lytic cycle activation of EBV were highlighted. This dual therapy synergistically induced killing of a panel of NPC cell lines and suppressed the growth of NPC xenografts in nude mice. Plus, BTZ limited SAHA's induction of EBV replication and hindered the production of infectious viral particles in NPC cells (Li et al., 2021[85]). In another study, Jiang et al. (2017[66]) studied the mechanism by which immune evasion affects the response to treatment of NPC. BTZ downregulated IFNγ-induced IDO expression via inhibition of JAK/STAT1 signaling pathway and promoted IkB-α phosphorylation-ubiquitination to release NF-kB from IkB-α (Guo et al., 2021[51]).

### Breast cancer

The primary treatment of breast cancer depends mainly on several chemotherapy combinations. However, unsuccessful treatments and poor prognostic outcomes are still being reported. This indicates the need to use targeted therapies such as proteasome inhibitors. By the start of 2000s, proteasome inhibitors were considered novel promising compounds in multiple hematological malignancies and solid tumors, based on the preliminary investigations seen in MM (Hamadneh et al., 2021[53]). However, following trials downgraded the incorporation of a proteasome inhibitor in solid tumors, hence minimizing the focus on proving this hypothesis (Keefe et al., 2013[72]). A recent study tested the application of PIs on some triple-negative breast cancer cell lines (TNBCs) which were able to survive after being exposed to standard doses of BTZ or carfilzomib which can potentially block the b5 peptidase of the proteasome (Meng et al., 2020[103]). While at higher (PIs) concentrations, cellular deterioration was achieved through the additional inhibition of b1 and b2, valorizing the necessity to act all sites of action to achieve a successful response. At this stage, CRISPR gene editing approaches were implemented to prove the complete inactivation of b1 and b2 re-sensitized cells to BTZ and carfilzomib, both *in vitro* and *in vivo* (Zhou et al., 2020[173]). In parallel, Radhakrishnan et al. (2010[123]) identified the source of the acquired resistance mechanism toward PIs where a transcription factor Nrf1 upregulated a set of proteasome subunit genes, hence increasing the proteasome content and its defensive action (Tapia-Laliena et al., 2019[146]). In fact, as cancer is known to be complex in nature, a partial response to therapy is not enough. Thus, Weyburne et al. (2017[159]) confirmed the limited ability of PIs to be used as individual therapy in solid tumors treatment as their potential to limit b2 activity is quite absent. So, PIs must be accompanied with a specific b2 inhibitor in order to obtain effective response (Martin et al., 2019[99]; Zhao et al., 2016[172]). From a medicinal chemistry perspective, structuration of a dual b2/b5 inhibitor is a challenging path. For that, other research groups attempted to block sites upstream of the proteasome, such as E1 and E3 enzymes as well as p97 resulting in an incomplete solid tumor response (Hui et al., 2013[59]; Jiao et al., 2019[67]; Yarza et al., 2021[165]). 

## BTZ Use in Liquid Tumors

### Mantle cell lymphoma (MCL)

Mantle cell lymphoma (MCL) is a rare, B cell non-Hodgkin's lymphoma with highly heterogeneous clinical presentation and aggressiveness (Jiang et al., 2017[66]). The inhibitory mechanism of proteasome exhibited MCL cell death through decreased NF-kappaB signaling and prevented IκB degradation and arrested cell cycle by limiting p27 breakdown while releasing high levels of reactive oxygen species (Fisher et al., 2006[39]; Hanel and Epperla, 2020[54]). BTZ was the first proteasome inhibitor to be FDA approved for relapsed/refractory MCL based on PINNACLE trial's findings (Goy et al., 2009[48]). Carfilzomib also showed promising effects in MCL with a lower encountered neurotoxicity compared to BTZ but the cardiotoxicity risk was more severe (Zhang et al., 2013[168]). Thus, BTZ is considered the unique proteasome inhibitor currently used with proven single-agent activity in relapsed/refractory MCL. Yet the efficacy of PIs in MCL cases is still based on their usage as combination therapies (Shah et al., 2018[134]). A brand new phase II randomized trial tested BTZ for its tolerability and efficacy either as a consolidation (BC) or maintenance (BM) therapy following immune-chemotherapy then autologous transplant (ASCT). Findings showed 54.1 % versus 64.4 % of 8-year PFS in BC and BM, respectively. Suggesting the promising value of BTZ in MCL practice (Lee et al., 2019[81]). While a phase II HOVON trial was based on administering BTZ as a maintenance therapy after R-CHOP, cytarabine and autologous stem cell transplantation in newly diagnosed patients with mantle cell lymphoma proved no added value for BTZ based on 63 % 5-year event free survival (EFS) versus 60 % 5-year (EFS) in BTZ and placebo group, respectively. Also, a 90 % 5 year overall survival (OS) was achieved in both groups (Kaplan et al., 2020[70]). These contradictory conclusions obtained impose the need for future improved hypothesis concerning BTZ use in MCL treatment regimens.

### Leukemia

Acute lymphoblastic leukemia (ALL) is the most frequently diagnosed cancer in children (Doorduijn et al., 2020[37]). The combination of BTZ, rituximab, and a pediatric-inspired ALL regimen was seen to be active and well tolerated in *de novo* CD20+ Philadelphia-negative precursor B-ALL (Hunger and Mullighan, 2015[60]). In children with relapsed or refractory ALL, the addition of BTZ to re-induction chemotherapy that includes mitoxantrone exhibited a complete response in the majority of cases with minimal toxicity occurrence (August et al., 2020[6]). Also, children and young adults with Early T-Cell Precursor (ETP) Acute Lymphoblastic Leukemia suffered from poor prognosis compared with other T-ALL subtypes. At this stage, Venetoclax was used as a sole therapy until resistance cases emerged (Jain et al., 2021[63]). Venetoclax was initiated individually with successful preliminary antitumor activity reaching resistant cases (Bose et al., 2017[16]). In support of this hypothesis, recent research work demonstrated that BTZ can induce the BH3-only protein NOXA responsible to downregulate, among other Bcl-2 protein family members, myeloid cell leukemia-1 (Tahir et al., 2017[142]). In addition, BTZ successfully modulated the oncogenic T-ALL driver NOTCH1 by suppressing the expression of Notch and its target genes (HES1, GATA3, and RUNX3) in parallel to a synergistic activity with dexamethasone, leading to a near-complete remission of T-ALL xenografted tumors *in vivo* (Bassan et al., 2018[10]). These results strengthened the promising objectives of implementing a combination of less-toxic bioactive drugs for patients with highly resistant R/R T-ALL. Hence, venetoclax along with BTZ (VEBO) imposed an effective and well-tolerated chemotherapy-free strategy for R/R T-ALL, including ETP especially, as a bridge to transplantation in fit patients (Li et al., 2019[89]). In another clinical trial, BTZ utilization post-induction chemotherapy with the ALL REZ BFM 95/96 was associated with an immediate treatment efficacy significantly higher in patients treated with BTZ compared to the control group (85.7 % vs 57.6 %), respectively (Follini et al., 2019[40]). Assessment of BTZ efficacy in terms of improved survival in children with T-Cell Lymphoblastic Lymphoma (T-LL) was also studied. The endpoints of standard risk (SR) and intermediate risk (IR) patients with T-ALL and T-LL treated with BTZ were successfully achieved through an improved 3-year EFS and OS. Therapy intensification allowed elimination of cranial radiation (CXRT) in the majority of patients without excessive relapse (Batmanova et al., 2017[11]). Thus, proving the importance of BTZ in standard therapy for *de novo* T-LL appears advantageous. The implementation of BTZ in Plasma Cell Leukemia (PCL) as an induction therapy in combination with chemotherapy exhibited a 79 % overall response rate (ORR) (Jurczyszyn et al., 2018[69]; Teachey et al., 2020[147]). Its success was also maintained throughout Hematopoietic cell transplantation (HSCT) with an extended 14.3 months of median progression-free survival (PFS) and 15.7 months median overall survival (OS) (D'Arena et al., 2012[31]; Mahindra et al., 2012[94]; Royer et al., 2016[131]). Following transplantation, applying BTZ during maintenance phase showed its safe and feasible ability to lower risk of graft-versus host-disease (GvHD) as well as impairing the activation of T-cells and antigen-presenting cell (Alsina et al., 2014[4]; Jakubowiak et al., 2011[64]; Katodritou et al., 2018[71]; Kneppers et al., 2011[76]; Neben et al., 2012[115]; Nencioni et al., 2006[116]; Sonneveld et al., 2012[137]).

## Promising Newly Discovered Pharmacological Effects of BTZ

BTZ therapeutic efficacy was not only limited to cancer tumors (Blanco et al., 2006[15]). In fact, BTZ was able to perform novel mechanisms of actions in terms of membranous nephropathy (MN), pulmonary hypertension (PH), neurofibromatosis and skin cancer (see Table 2(Tab. 2); References in Table 2: Chou and Talalay, 1984[24]; Cooper and Giancotti, 2014[29]; Fisher et al., 2006[39]; Geara et al., 2021[45]; Kim et al., 2012[74]; Lee et al., 2021[82]; Li et al., 2010[88], 2012[86], 2014[87]; Liu et al., 2021[90]; Meeth et al., 2016[100]; Nakagawa et al., 2000[113]; Synodos for NF2 Consortium et al., 2018[141]; Thompson, 2013[148]; Voltan, 2018[155]; Yalon et al., 2013[163]; Zhang et al., 2016[169]; Zhou and Hanemann, 2012[174]).

## Conclusion

BTZ use is mainly approved for retreating patients with MM and as a first-line option for patients with mantle-cell lymphoma. Consequently, the prognostic impact of BTZ in patients with other liquid tumors and solid tumors is still under investigation. In our review, we detailed the conventional and advanced BTZ mechanisms of action which allowed BTZ to be a crucial option of MM anti-tumor regimen. Moreover, BTZ succeeded to exhibit its therapeutic potential in different types of solid and liquid tumors. Specifically, BTZ expressed better therapeutic efficacy in ovarian, endometrial and nasopharyngeal cancers. However, further investigations should be executed to adequately assess the promising value of BTZ in order to reach successful treatment outputs and better patient's prognosis. Furthermore, this review highlighted the newly discovered pharmacological effects of BTZ in multiple diseases such as membranous nephropathy, pulmonary hypertension and neurofibromatosis, which accredited BTZ with additional clinical value in cancer and other illnesses.

## Notes

Mohammad Alwahsh and Joviana Farhat contributed equally as first author.

## Declaration

### Author contributions

Conceptualization, MA, JF, and RH; methodology, MA, JF; writing-review and editing, MA, JF, ST and LH; supervision, MA, LH and RH. All authors have read and agreed to the published version of the manuscript.

### Funding

Not applicable.

### Institutional review board statement

Not applicable.

### Informed consent statement

Not applicable. 

### Acknowledgments

All authors acknowledge their respective institutions for their support. 

### Conflicts of interest

The authors declare no conflict of interest.

## Figures and Tables

**Table 1:**
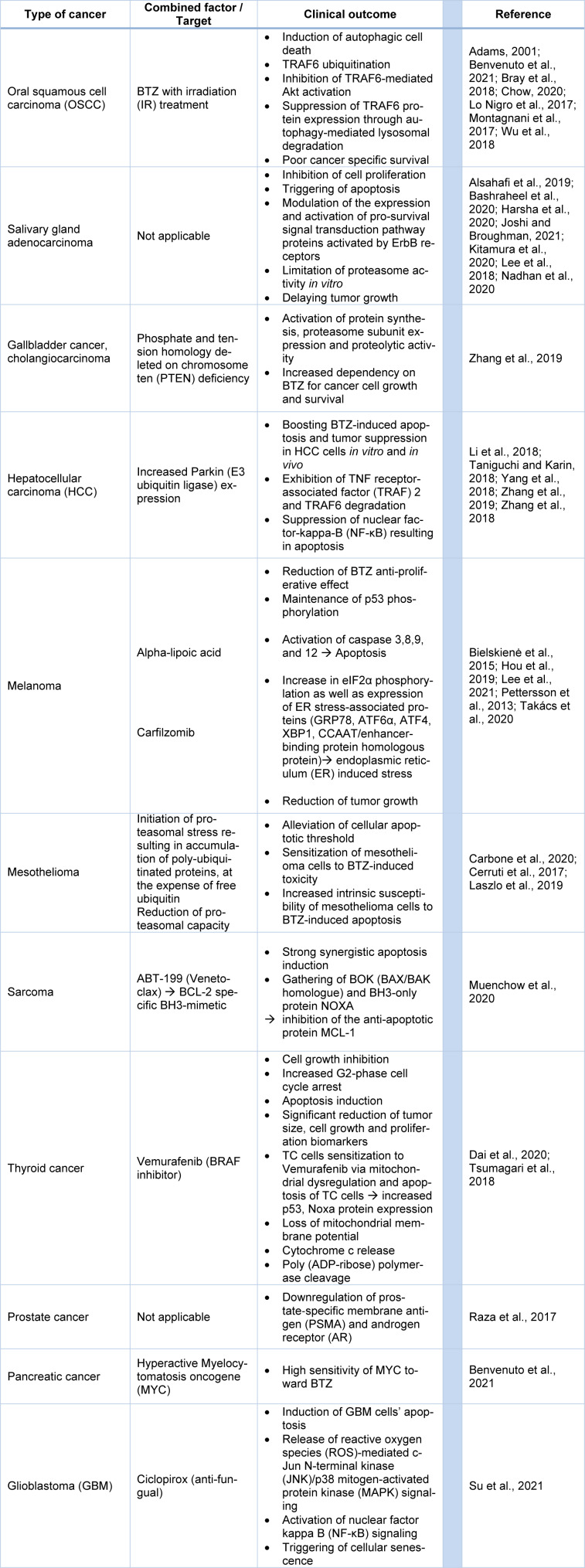
BTZ efficacy in other solid tumors

**Table 2:**
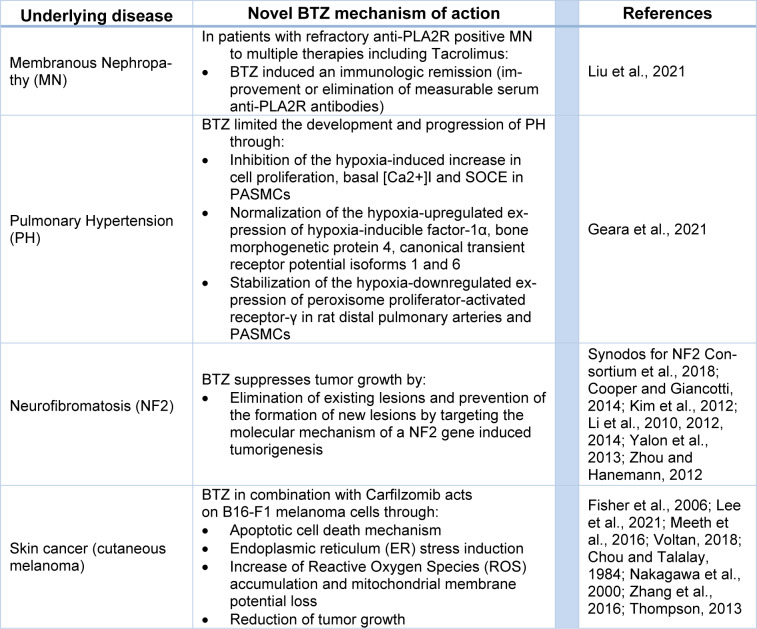
Novel BTZ application in multiple diseases
